# Living Without Temporal Cues: A Case Study

**DOI:** 10.3389/fphys.2020.00011

**Published:** 2020-02-07

**Authors:** Maria-Angeles Bonmati-Carrion, Victoria L. Revell, Tom J. Cook, Thomas R. E. Welch, Maria-Angeles Rol, Debra J. Skene, Juan Antonio Madrid

**Affiliations:** ^1^Chronobiology Laboratory, Department of Physiology, University of Murcia, IMIB-Arrixaca, Espinardo, Spain; ^2^Ciber de Fragilidad y Envejecimiento Saludable (CIBERFES), Madrid, Spain; ^3^Surrey Sleep Research Centre, Faculty of Health and Medical Sciences, University of Surrey, Guildford, United Kingdom; ^4^Windfall Films, London, United Kingdom; ^5^Chronobiology, Faculty of Health and Medical Sciences, University of Surrey, Guildford, United Kingdom

**Keywords:** circadian, time isolation, wrist temperature, mid-sleep, performance, phase advanced, dim light, clock-time estimation

## Abstract

Isolation from external time cues allows endogenous circadian rhythmicity to be demonstrated. In this study, also filmed as a television documentary, we assessed rhythmic changes in a healthy man time isolated in a bunker for 9 days/nights. During this period the lighting conditions were varied between: (1) self-selected light/dark cycle, (2) constant dim light, and (3) light/dark cycle with early wake up. A range of variables was assessed and related to the sleep-wake cycle, psychomotor and physical performance and clock-time estimation. This case study using modern non-invasive monitoring techniques emphasizes how different physiological circadian rhythms persist in temporal isolation under constant dim light conditions with different waveforms, free-running with a period (τ) between 24 and 25 h. In addition, a significant correlation between time estimation and mid-sleep time, a proxy for circadian phase, was demonstrated.

## Introduction

Keeping our physiological and behavioral processes temporally organized is essential for maintaining a good state of health and wellbeing. The circadian timing system needs to be reset every day since its endogenous period (τ) is not exactly 24 h (Middleton et al., 1996; Hiddinga et al., 1997; Carskadon et al., 1999; Czeisler et al., 1999; Burgess and Eastman, 2008). Thus, our circadian system utilizes cyclic environmental cues (*zeitgebers*) such as the light/dark cycle to synchronize the central pacemaker located in the suprachiasmatic nuclei (SCN) on a daily basis and remain entrained to the 24 h day. The circadian system will free-run (oscillate with its endogenous period) in constant conditions.

Variables that have been shown to oscillate in a circadian manner in humans include core and skin body temperature (Aschoff, 1983; Carskadon et al., 1999; Wyatt et al., 1999; Hanneman, 2001), melatonin (Lockley et al., 1997; Dijk et al., 1999; Zeitzer et al., 1999) and cortisol (Linkowski et al., 1993; Skene et al., 1999; van de Werken et al., 2014) production, blood pressure (Shea et al., 2011), sleep (Aschoff, 1993; Dijk and Czeisler, 1995; Wyatt et al., 1999), subjective sleepiness (Wu et al., 2015), and mood (Koorengevel et al., 2000; Murray et al., 2002, 2009; Wirz-Justice, 2003). Other variables that exhibit circadian rhythmicity are those related to intellectual or cognitive performance, such as concentration, reaction time, memory, etc. (reviewed in Dijk et al., 1992; Jewett et al., 1999; Schmidt et al., 2007; Wu et al., 2015), as well as other aspects related to physical performance (reviewed in Teo et al., 2011). Another rhythmic variable that has rarely been studied is the perception of time, although early work showed that the ability to estimate short (5–10 s) time intervals fluctuated throughout the day (Aschoff, 1998).

Adequate exposure to *zeitgebers* is essential for synchronization with the external environment but also internally to ensure a normal phase relationship among these rhythmic variables. However, nowadays, a high percentage of the population are not exposed to a strong light/dark cycle either spending the majority of their time indoors with low light contrast between day and night (Roenneberg et al., 2003), or performing shift work (Straif et al., 2007). Shift work and the associated circadian misalignment and shortened sleep has been linked with a short term impact on performance, productivity and safety as well as a long term impact on health (Möller-Levet et al., 2013; Eckel et al., 2015), predisposing people to chronodisruption (reviewed in Bonmati-Carrion et al., 2014) due to the misalignment between social time (including work schedule and leisure time), behavior (sleep/wake, feeding/fasting) and internal biological time (endogenous circadian phase). Chronodisruption has been associated with a higher prevalence of different health impairments, including metabolic syndrome (Garaulet and Madrid, 2010; Reiter et al., 2011), cardiovascular disease (Knutsson and Bøggild, 2000), cognitive impairment (Cho et al., 2000) and different types of cancer [breast (Davis et al., 2001; Schernhammer et al., 2001), colorectal (Schernhammer et al., 2003) and prostate (Conlon et al., 2007; Kubo et al., 2006)].

Protocols and tools to study endogenous circadian rhythmicity and entrainment are continuously being developed. In the 1930s, Nathaniel Kleitman was the first to explore the endogenous nature of circadian rhythms in humans (Kleitman et al., 1938). In 1965, Jürgen Aschoff, another pioneer in the study of biological rhythms, extended the study of the circadian timing system in humans (Aschoff, 1965). Volunteers lived in a bunker (in groups or individually) for long periods of time, without access to any external temporal cues to synchronize their internal clocks, although in some experiments participants could self-select their light/dark cycle (Aschoff, 1965, 1967). This, and subsequent experiments, confirmed the endogenous origin of human circadian rhythms, establishing one of the principles of circadian biology (Aschoff, 1967). Isolation studies were also performed by Pöppel and Giedke, who also explored diurnal variation of time perception (Pöppel and Giedke, 1970). More recent experiments on healthy volunteers in continuous dim light (Middleton et al., 1996, 1997) and blind people with no perception of light (Lewy and Newsome, 1983; Lockley et al., 1997, 2007; Skene and Arendt, 2007) have also allowed free-running circadian rhythms in humans to be characterized. All of these more recent experiments, however, have included social contact and knowledge of clock time.

Another approach to study the periodicity of human circadian rhythms is the forced desynchrony (FD) protocol (Minors and Waterhouse, 1981; Schmidt et al., 2007; Stack et al., 2017), pioneered by Kleitman (1987). This protocol exposes participants to a “day” that is significantly longer (e.g., 28, 40 h) or shorter (e.g., 20 h) than the 24-h day. Since the imposed daylength is outside the range of entrainment (the clock cannot be synchronized to these daylengths), circadian rhythms free-run at their endogenous period (τ). This protocol, however, also normally involves social contact between participants and/or researchers. Therefore, an experiment under constant dim light conditions with social isolation and no knowledge of clock time would provide additional insight into human circadian rhythmicity.

Although this case study was originally designed as part of a television documentary (Body Clock: What makes us tick?) that aimed to disseminate chronobiology knowledge and research to the general public, the unique social and time isolation, and dim light conditions in the bunker, as well as the continuous physiological monitoring that occurred, provided an excellent opportunity to study the human circadian clock while revisiting the first bunker experiments mentioned above. In addition, the chronodisruption conditions experienced during the light/dark cycle with early wake up (third stage, involving an abrupt curtailment of sleep) is not often performed under temporal and social isolation.

The scientific objective of the protocol was to study rhythmic changes in a person living in social/environmental isolation over three conditions (baseline light/dark cycle, constant dim light and a light/dark cycle with early wake up). Wrist temperature, motor activity and light exposure were assessed, and the sleep-wake cycle derived, using a novel ambulatory monitoring (ACM) system (Kronowise) (Madrid-Navarro et al., 2019). Another aim of the study was to evaluate the estimation of clock time, a variable rarely assessed, in parallel with assessments of subjective sleepiness, reaction time, memory/concentration, grip strength, sprint time and mood. We hypothesized that most of the physiological variables measured would follow a circadian variation, tending to free-run (exhibit non-24 h periodicity) under constant conditions. We also expected an acute impairment of waking performance following the light/dark cycle with early wake up.

## Materials and Methods

### Participant

The participant was a healthy, non-smoking, 40-year-old male who was invited to participate in the BBC television documentary study (Cook, 2018). Full details of the study protocol and procedures were explained to the participant prior to the start of the study and he was provided with a written document describing the study protocol. This document explained the different stages he would undergo but not exactly when they would happen. The participant was aware he could withdraw from the experiment at any time and written informed consent was obtained. The participant also underwent an independent psychological assessment to ensure he was a suitable subject for the experiment. All research protocols were approved by the BBC’s compliance department, who reviewed the experiment design and ethical issues. All research was performed in accordance with relevant guidelines and regulations. A full risk assessment was carried out by the documentary producers, that was reviewed by an independent external risk assessment company (First Option – health and safety specialists who advise the television industry). The participant was also offered follow up support if needed.

### Protocol

Throughout the 10 day study, the participant remained in a bunker with no direct human contact and no knowledge of actual clock time (i.e., no telephone, internet, television, radio, or natural light). The experiment consisted of three consecutive phases: self-selected light/dark cycle (Stage 1), constant dim light (Stage 2) and light/dark cycle with early wake up (with consequent sleep curtailment) (Stage 3). The experiment was performed in May–June 2018 in South West England.

**FIGURE 1:**
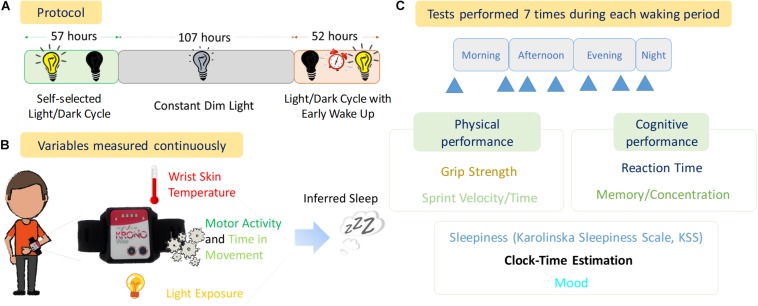
Experimental Design. **(A)** Protocol of the experiment with the duration of each stage (57 h, Self-selected Light/dark cycle; 107 h, Constant Dim Light; and 52 h, Light/dark cycle with early wake up), **(B)** variables measured continuously throughout the experiment, **(C)** diagram of tests performed and the seven approximate times (blue arrows) within each waking period.

The experimental phase is shown in Figure 1 and consisted of:

#### Stage 1

Self-selected light/dark cycle (57 h, 2.4 days). Light sources were a mixture of bright LED panels and halogen desk lights (color temperature approximately 3200 K). When the lights were on, this resulted in similar brightness to a well-lit indoor room at night. When the lights were off, the room was completely dark (0 lux). A bright infra-red light source was present in the bedroom to allow infra-red video recordings to be obtained in the 0 lux conditions. The light switch was controlled remotely by the research team, but the participant chose when he wanted the lights on or off.

#### Stage 2

Dim light phase (107 h, ∼4.5 days). Light sources were set to be <10 lux throughout the bunker using primarily dimmed halogen lamps (3200 K). This light level was maintained constantly from Day 3 of the experiment at 19:10 h (local time) until Day 8 (Night 7) at 06:00 h. Again, infra-red lights were used in several rooms to provide illumination for infra-red cameras. If needed, the participant also wore a very dimly lit head torch to help him get around the darkest areas of the bunker.

#### Stage 3

Light/dark cycle with early wake up, comprised the re-introduction of a light/dark cycle with a forced early awakening (52 h, ∼2.2 days). To simulate an early start shift work schedule the volunteer was awoken at 06:00 h local time on Nights 7–9 with a telephone call and the researchers turning on the room lights. On Night 7 this was approximately 3.5 h into his sleep period (sleep restriction). Following this he was then free to choose his activities including when to go to sleep (when he was ready, he asked the team to turn off the lights).

Throughout the study, the participant remained in isolation with no time cues. Apart from a brief phone call at the beginning of Stages 2 and 3 with a researcher, he had no verbal or visual contact with the team. He was free to choose the timing of all his activities (sleep/wake, meals, exercise etc.) throughout the study (except being woken up early in Stage 3). Upon request, he was provided with ready-made meals of his choice up to the daily total Kcal recommended for an adult male. He controlled his own meal times, as well as the times when he performed the tests.

The participant had no access to any light-emitting devices apart from a small camera with the screen (2 × 4 cm, 2° 51′ 0.85^″^ visual angle) brightness set at the minimum. This was used for self-filming and to record his feelings throughout the study. He also had some paper books and he could exercise by using weights, press ups, and running up and down the corridor.

### Ambulatory Circadian Monitoring

The participant continuously wore a small, watch-like device for Ambulatory Circadian Monitoring, “Kronowise 3.0” (Kronohealth SL, Spain) (Arguelles-Prieto et al., 2019) on his non-dominant hand to reduce possible masking of motor activity on the measured variables.

Wrist skin temperature, light exposure (in three spectral bands: visible (400−700 nm), short-wavelength between 460–490 nm and infrared >800 nm) and triaxial motor acceleration were continuously recorded. From triaxial acceleration, (i) acceleration of movement, (ii) wrist posture (tilt of the *x*-axis) and (iii) time in movement were assessed. Time in movement was calculated as the time, in periods of 0.1 s, in which a movement in any of three axes was detected, this information being particularly useful to discriminate between sleep and wake states (Madrid-Navarro et al., 2019). The sample rates were 1 Hz for wrist skin temperature and light exposure, 10 Hz for acceleration and 0.033 Hz (1 reading per epoch) for wrist position. The data were then processed and saved into 30 s epochs throughout the experiment.

Communication with the ACM Kronowise device was established using Kronoware 10.0 software (Kronohealth SL, Spain) via a USB port. This software allows visual inspection of the data before analysis to eliminate artifacts and the calculation of basic circadian and sleep parameters. Data were converted into a text file to be analyzed by the chronobiological software “Circadianware,” implemented on the on-line Kronowizard platform^1^ (University of Murcia).

#### Sleep Detection

The sleep parameters (sleep onset, offset, duration) and light-associated parameters (lights off related to when the volunteer prepared for sleep and lights on related to wake up) were estimated using the Kronowizard website (see text footnote 1, University of Murcia) and based on the TAP (wrist Temperature, motor Activity and body Position integrated variable) algorithm (Ortiz-Tudela et al., 2010). Thus an epoch was scored as sleep when TAP was under a default threshold (0.28), previously validated by polysomnography (PSG) (Ortiz-Tudela et al., 2014). Mid-sleep time, derived from the Kronowise data, was used as the estimation of circadian phase.

### Performance, Subjective Sleepiness and Mood Tests

The participant completed a variety of performance tests at self-selected times throughout each day of the study. The variables tested included reaction time (Fitlight Trainer^TM^, FITLIGHT Corp., Denmark), memory/concentration [paper-based digit symbol substitution test, DSST (Rosano et al., 2016)], grip strength (Camry 200 lbs/90 kgs Digital Hand Dynamometer, Camry^®^ Scale, United States) and sprint time over a distance of 11 meters (TC Timing Systems, Brower Timing^©^). In addition, subjective sleepiness [Karolinska Sleepiness Scale (Akerstedt and Gillberg, 1990)] and mood [assessed using 4 visual analog scales 1–9 (Lockley et al., 2008; Revell et al., 2006) ranging from very alert (1) – very sleepy (9), very cheerful (1) – very miserable (9), very calm (1) – very tense (9), very elated (1) – very depressed (9)] were assessed.

The participant was asked to perform seven sets of tests each waking day. He was required to do the first one as soon as he woke up; the second, during the middle of his subjective morning, followed by the third one before lunch. The next one needed to be completed during the middle of his afternoon, and the last three sets were required to be done before dinner, during the middle of his evening and just before going to bed, respectively. Three attempts were recorded for each test at each time point and the mean calculated.

### Estimation of Clock Time

At each test time for performance, subjective sleepiness and mood assessments, the participant was asked “What time is it?” Clock times (both actual and estimated) were converted into time (h) since wake up (time zero) and the difference between them calculated. In order to correlate with the midpoint of sleep, the differences between actual and estimated times were averaged for each study day.

### Analyses

Each variable recorded by Kronowise (Kronohealth SL, Spain) in 30 s epochs was averaged hourly to be able to compare these data with the performance test data. Mid-sleep time was calculated as the midpoint of the sleep period as detected by the KronoWare (Kronohealth SL, Spain) software.

To calculate the period of ACM variables during Stage 2 (constant dim light), El Temps software (version 1.228, copyright Diez-Noguera, University of Barcelona) was used. For non-ACM variables, period was calculated by averaging the time between maximum values throughout that stage.

To calculate amplitude or difference between day and night, mean waveforms were calculated (not shown) for each variable according to the inferred sleep onset for the preceding night, considering time as “*Hours After Sleep Onset*” (HASO). Each variable was averaged from 00:00 to 08:00 h (HASO) for night time and from 09:00 to 23:00 h (HASO) for daytime and then the difference between them was calculated.

#### Statistics

Averaged data from the different days within each experimental stage are expressed as mean ± SD.

Correlations were also performed between variables recorded by Kronowise and the performance tests results. All calculations and statistical analyses were performed using SAS version 9.4.

## Results

**FIGURE 2:**
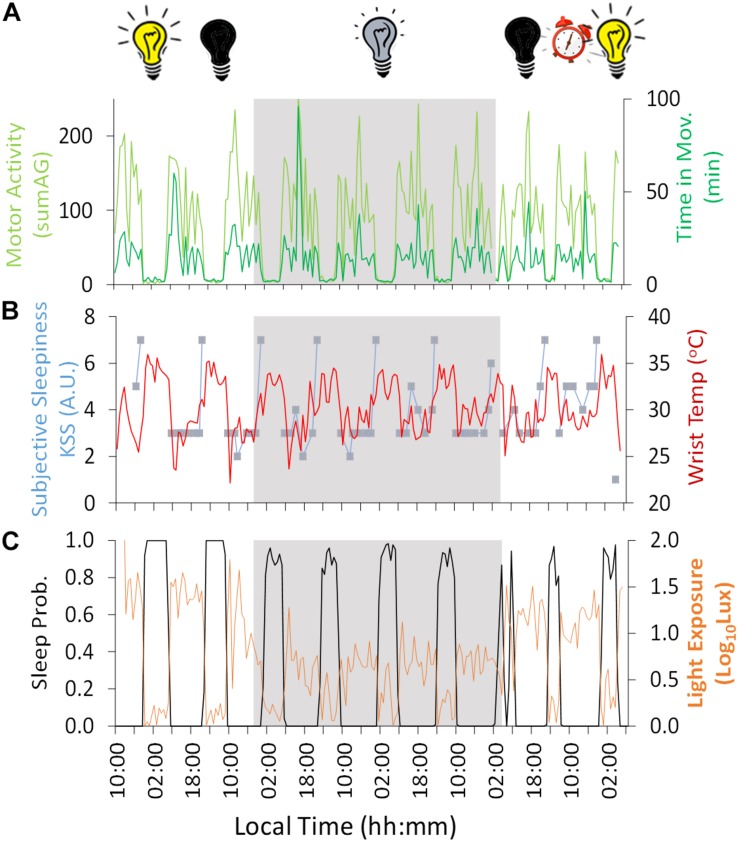
Ten-day ambulatory recording of **(A)** time in movement (light green) and motor activity (dark green), **(B)** wrist temperature (red) and subjective sleepiness (Karolinska Sleepiness Scale, KSS) (blue), **(C)** sleep probability (black) and light exposure (orange). Gray areas indicate Stage 2 (constant dim light). Previous and subsequent days correspond to Stage 1 (self-selected light/dark cycle) and Stage 3 (light/dark cycle with early wake up), respectively. Black bulb indicates darkness, yellow bulb indicates bright light (Stage 1, self-selected light/dark cycle), gray bulb indicates dim light (Stage 2) and alarm clock indicates light/dark cycle with early wake up (Stage 3).

The conditions of this experiment allowed us to demonstrate circadian rhythmicity in a range of variables (Figure 2) and to calculate the free-running periodicities (τ) of these variables during the constant dim light conditions (Stage 2: 107 h, ∼4.5 days): wrist temperature (τ 24.16 h), motor activity (τ 25.16 h and 24.30 h for acceleration and time in movement, respectively), sleep-wake cycle (inferred from the novel ACM system: midpoint of sleep, τ 24.40 h), subjective sleepiness and mood (τ 24.80 h).

Regarding the variables measured by Kronowise^®^, motor activity (Figure 2A) – both acceleration and time in movement– showed a diurnal pattern, with higher values during the day across the experiment, with a period (τ) in constant dim light of 25.16 and 24.30 h, respectively. Wrist skin temperature (Figure 2B) showed the inverse pattern, with higher values during the night (reaching a maximum of approximately 34°C, 32.9 ± 0.3°C) and lower values during the day (minimum ≈ 27°C, 28.8 ± 0.6°C), showing a period (τ) of 24.16 h during the constant dim light phase. The amplitude of this diurnal rhythm was reduced by 77% during the dim light conditions (Stage 2) compared to the self-selected light/dark cycle (Stage 1) and only seemed to recover during the second and last day of Stage 3 (light/dark cycle with early wake up).

Light exposure recording (Figure 2C) during Stage 1 (self-selected light/dark cycle) confirmed that environmental light levels exhibited higher values during the day (maximum reaching around 100 lux) and darkness during the night. During Stage 2, the 4-day dim light phase (beginning on Day 3 at 19:10 h, local time), the experimental conditions were confirmed as being continuously <10 lux except in two epochs (not exceeding 20 lux). During Stage 2, when the light was constantly dim (<10 lux), the wrist worn monitor showed lower light levels during the night than during the day (with a mean reduction of approximately 65%), probably because the participant covered himself with a blanket, also covering the light sensor during part of the subjective night.

**FIGURE 3:**
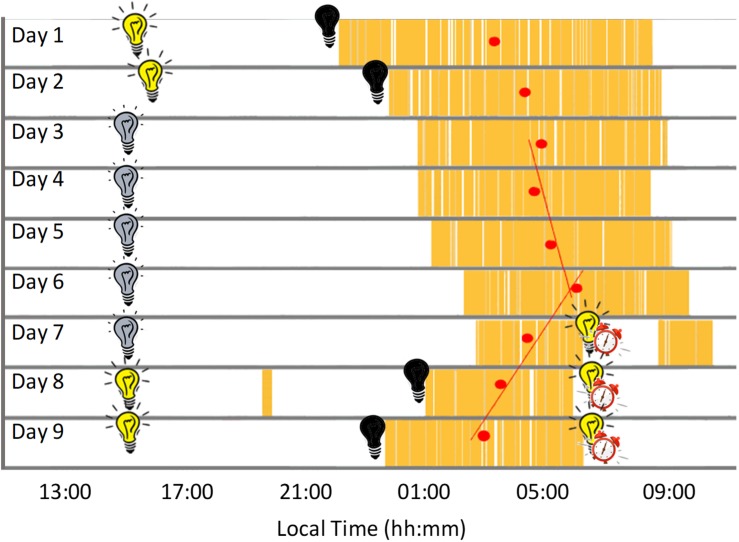
Actogram built from the daily records showing sleep periods in yellow and mid-sleep time (red circles) for each night. Black bulb indicates darkness, yellow bulb indicates bright light (Stage 1, self-selected light/dark cycle), gray bulb indicates dim light (Stage 2) and alarm clock indicates light/dark cycle with early wake up (Stage 3).

During Stage 1 (self-selected light/dark cycle) the sleep-wake pattern inferred from the ACM device (Figures 2C, 3 and Table 1) showed a delay from Night 1 to 2 in sleep onset (104 min), midpoint of sleep (64 min) and sleep offset (23 min). During the dim light conditions (Stage 2) the midpoint of sleep delayed on average 23 ± 28 min (mean ± SD) each night, with a period (τ) of 24.40 h (Nights 2–6).

**TABLE 1:** Summary of estimated sleep and associated light exposure.

Night	Lights off	Sleep onset	Sleep offset	Lights on	Sleep duration	Mid-sleep point
1	21:52	22:05	08:30	08:39	10:25	03:17
2	23:40	23:49	08:53	09:19	09:04	04:21
3	DL	00:45	09:01	DL	08:16	04:53
4	DL	00:47	08:28	DL	07:41	04:37
5	DL	01:14	09:14	DL	08:00	05:14
6	DL	02:12	09:53	DL	07:41	06:02
7	DL	02:43	06:10*	06:10^$^	03:27^#^	04:26
8	00:50	01:02	05:54*	06:01^$^	04:52	03:28
9	23:02	23:39	06:13*	06:23^$^	06:34	02:56

Lights off/on and sleep onset/offset times, nocturnal sleep duration and mid-sleep point (all derived from Kronowise ACM) each day of the study (expressed as hh:mm). DL is continuous dim light and, thus, lights off/on is not applicable during Stage 2. *Sleep offset was curtailed on those days. ^#^Volunteer took a morning nap that day, thus the total sleep duration would be increased (Table showing only night sleep). ^$^Indicates enforced lights on.

Sleep duration (Table 1 and Figure 3), derived from the ACM device, tended to be longer (9.7 ± 0.7 h) at the beginning of the study (self-selected light/dark cycle), when the participant was entirely free to choose the time to go to sleep and to wake up (no alarm, no daylight cycle, no time cues), tending to be shorter during the dim light phase (7.9 ± 0.2 h).

**FIGURE 4:**
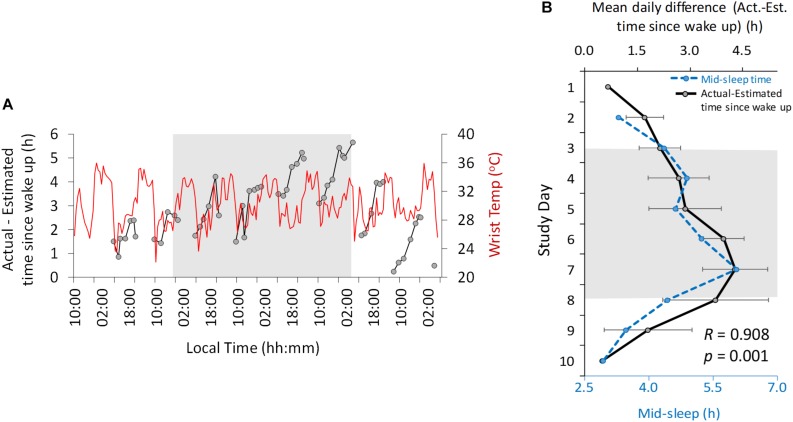
Ten-day ambulatory recordings of wrist temperature rhythm (red) plotted with the difference between actual and estimated time since wake up (black, **A**). Daily mean difference (± SD) between actual and estimated time since wake up (**B**, solid line, black) with mid-sleep time (dashed line, blue). Gray area indicates Stage 2 (constant dim light). Previous and subsequent days correspond to Stage 1 (self-selected light/dark cycle) and Stage 3 (light/dark cycle with early wake up), respectively.

**FIGURE 5:**
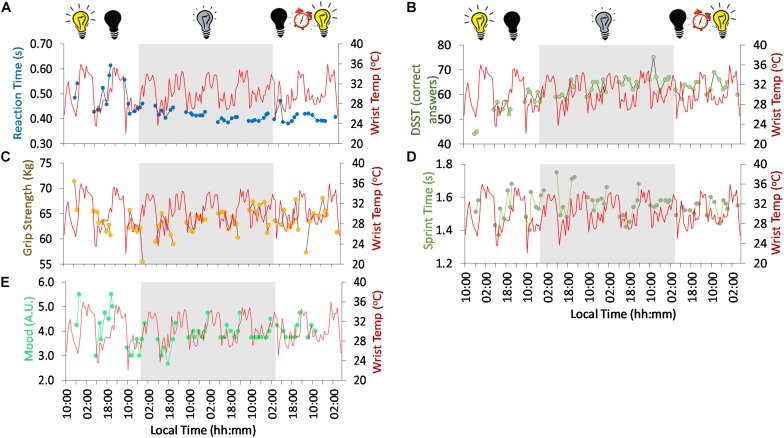
Ten-day ambulatory recordings of wrist temperature rhythm (red) plotted with reaction time (**A**, blue), digit symbol substitution test (DSST) (**B**, green), grip strength (**C**, brown), sprint time (**D**, green), mood (higher values, more depressive mood) (**E**, turquoise). Gray areas indicate Stage 2 (constant dim light). Previous and subsequent days correspond to Stage 1 (self-selected light/dark cycle) and Stage 3 (light/dark cycle with early wake up), respectively. Black bulb indicates darkness, yellow bulb indicates bright light (Stage 1, self-selected light/dark cycle), gray bulb indicates dim light (Stage 2) and alarm clock indicates light/dark cycle with early wake up (Stage 3).

Since of the variables measured, wrist temperature has the greatest endogenous component, as previously demonstrated through demasking procedures, this was plotted in parallel with subjective sleepiness (Figure 2B), time estimation (Figure 4), performance results and mood (Figure 5). Subjective sleepiness (KSS) (Figure 2B) was greatest immediately before bedtime, regardless of the experimental phase. The time of maximum sleepiness tended to delay from the beginning (23:05 h) toward the end of Stage 2 (1:29 h), with a period (τ) of 24.8 h during this dim light phase. Wrist skin temperature also appeared to be a good predictor of sleepiness score under constant conditions, showing a positive correlation (*R* = 0.486, *p* = 0.007) during the constant dim light conditions, but failing to do so during the self-selected light/dark cycle and light/dark cycle with early wake up (*R* = 0.318, *p* = 0.290 and *R* = −0.268, *p* = 0.334, respectively).

Another variable assessed in this study was time estimation (Figure 4). Throughout the study the actual local clock-time was always underestimated by the participant (i.e., he always thought it was earlier than it actually was). The difference between the actual time and estimated time (expressed relative to wake up time) oscillated with a diurnal and circadian pattern (Figure 4A) in Stages 1 and 2, respectively, showing a period of 25.42 and 24.28 h regarding maximum and minimum difference, respectively. The maximum difference occurred immediately before bed time and the minimum difference occurred in the morning.

The difference between the actual time and the estimated time since wake up increased progressively from the beginning of the study (mean 0.67 h difference, Stage 1) to the beginning (2.68 h) and end (4.29 h) of the dim light phase (Stage 2). This difference decreased again during Stage 3 (0.5 h at the end). This change in time estimation since wake up was strongly correlated (*R* = 0.908, *p* = 0.001) (Figure 4B) with the midpoint of sleep across all the study days.

Regarding psychomotor tests, both reaction time (Figure 5A) and digit symbol substitution tests (DSST, a psychomotor test) (Figure 5B) showed a tendency to improve throughout the study. Reaction time ranged on average from 0.49 ± 0.06 s at the beginning (self-selected light/dark cycle) to 0.41 ± 0.02 s at the end of the study, thus reflecting an improvement in reaction rate. The DSST scores also improved from the self-selected light/dark cycle (54.7 ± 5.4 right answers) to the dim light (63.5 ± 3.8 right answers) and light/dark cycle with early wake up (63.1 ± 2.7 right answers) phases.

Despite the tendency to improve across the study period, a clear diurnal pattern in reaction time was observed at the beginning of the study (self-selected light/dark cycle), with better performance in general (thus, shorter reaction times) during the day compared to the evening. However, from the beginning of the dim light conditions (Stage 2), the day-night difference was clearly dampened. During the 57 h light/dark cycle (self-selected light/dark cycle, Stage 1), reaction time was significantly correlated with the wrist temperature pattern (*R* = 0.513, *p* = 0.042), thus, slower reaction time with higher wrist temperature values.

Physical performance also followed a diurnal rhythm with both grip strength (Figure 5C) and sprint speed (Figure 5D) showing better results in the afternoon (∼5 h after wake). The time of maximum performance (greater grip strength and sprint velocity) tended to delay during the first 3 days of constant dim light (from 13:56 to 17:37 h, 221 min), advancing again on the subsequent days of dim light (back to ∼13:50 h). Both variables followed an inverse relationship with wrist temperature.

Mood (Figure 5E) also followed a diurnal pattern with a peak in depressive mood (higher scores) toward bedtime (21:23 h), and a minimum at the beginning of the day in the light/dark cycle (self-selected light/dark cycle, Stage 1). The time of maximum score tended to delay throughout the dim light conditions (Stage 2), from 23:05 h at the beginning of Stage 2 to 1:29 h at the end of Stage 2, indicating a period (τ) of 24.8 h. The peak time again advanced on the first day of the light/dark cycle with early wake up (Stage 3). These peak times in depressive mood are in agreement with those for maximum sleepiness (both patterns showed a strong and significant correlation, *R* = 0.644, *p* < 0.0001), indicating a clear relationship between mood and sleepiness. To assess the effect of the experimental protocol on mood, the mean (± SD) maximum depressive scores were calculated [5.50 ± 0.00, Stage 1; 4.53 ± 0.19, Stage 2; and 4.75, Stage 3 (*n* = 1)]. The minimum scores, however, tended to increase throughout the experiment Stage 1, 3.0 ± 0.0; Stage 2, 3.48 ± 0.47; and Stage 3, 3.75 (*n* = 1). Thus, there seems to be an effect of the absence of light/dark cycle on the amplitude of this rhythm, decreasing from ∼2.5 in Stage 1 (self-selected light/dark cycle) to ∼1.05 and ∼1 in Stage 2 (constant dim light) and Stage 3 (light/dark cycle with early wake up), respectively.

The phase advance light/dark cycle performed in this study involved waking up the participant at 06:00 h for 3 days, which was 3:43 h earlier than wake up time on the last day of the dim light conditions, thus advancing the mid-sleep time by 96 min. As expected, the participant slept around 3.5 h on the first night of the light/dark cycle with early wake up, which resulted in sleep curtailment and him having a morning nap that day. On the subsequent two nights, his sleep onset advanced, probably due to increased homeostatic pressure, with sleep durations of ∼5 and 6.5 h, respectively. As expected, there was a resultant phase advance in the majority of variables recorded. Following the forced early wake up (Day 8), there was a reduction in DSST score, grip strength and reaction rate (longer reaction times) (Supplementary Figure S1) in the first sampling session compared to the first sampling session the previous day. Sprint speed, however, did not show an acute impairment due to the early start simulation.

## Discussion

The design of this experiment, originally conceived as a television documentary (Cook, 2018), allowed us to study the internal circadian clock without any influence of external temporal cues (environmental or social). This permitted estimation of the endogenous period of a number of rhythmic variables, as well as changes in their amplitude and phase. Our findings also revealed a circadian modulation of clock-time estimation in constant dim light conditions. Clock-time estimation revealed that the participant always thought it was earlier than it actually was and this was likely related to his estimation of his sleep/wake time, since he reported sleeping from 23:30 to 06:00 h before starting the experiment (thus, earlier than in the bunker). Time estimation since wake up was correlated with the midpoint of sleep (a parameter that has been previously proposed as a marker of chronotype (Roenneberg et al., 2003; Roenneberg, 2012). In addition, the midpoint of sleep could be related to wake duration, which was previously reported by Aschoff (1998) to be related to time estimation in time-isolated volunteers. The effect of time and social isolation on time estimation has previously been described in volunteers, who tended to significantly overestimate 1-hour periods (Aschoff, 1985).

The circadian pattern found in time estimation is another remarkable finding of this study, since although this had previously been reported regarding short lapses (10 s) (Pöppel and Giedke, 1970; Kuriyama et al., 2005), to the best of our knowledge, this is the first time that the circadian variation in time estimation for longer periods (such as a whole day) has been described in humans, either in the presence of time cues or under constant dim light conditions. There is previous literature that links time perception with the circadian clock. In hamsters, for example, a tandem SCN and other circadian oscillators have been suggested to underlie time memory (Ralph et al., 2013). In our case study, the difference between actual and estimated time since wake up increased toward the end of the subjective day, and was lower in the early hours of the morning. This “resetting” effect of wake up could reflect the sleep feedback on the circadian system, acting as a *zeitnehmer*. As previously suggested (Danilenko et al., 2003) this *zeitnehmer* may not be strong enough to entrain other overt rhythms, but it may be effective enough to entrain time estimation. We also hypothesized that sleep offset/wake up time would be a stronger *zeitnehmer* than sleep onset, in accordance with the fact that in preindustrial societies awakening usually occurred before sunrise, thus anticipating it, while sleep onset started more than 3 h after sunset (Yetish et al., 2015). Early studies suggested a coupling between diurnal variation in time perception and the circadian oscillator, but they did not discard the influence of other rhythmic variables (such as body temperature) on time perception (Pöppel and Giedke, 1970). In our study, no significant correlations between estimated time since wake up and wrist skin temperature nor other measured variables were found.

The variables measured by the ambulatory device, Kronowise^®^, exhibited the expected previously described motor activity patterns, with higher values during the day and lower values during the night (Ortiz-Tudela et al., 2010; Bonmati-Carrion et al., 2013, 2015; Madrid-Navarro et al., 2018) and the opposite pattern for wrist temperature (Sarabia et al., 2008; Ortiz-Tudela et al., 2010; Bonmati-Carrion et al., 2013). The wrist temperature amplitude was reduced under constant dim light conditions which may be due to the absence of positive masking by light (Martinez-Nicolas et al., 2013). Light exposure recording served to confirm that the average light levels were significantly reduced during the dim light stage.

The ambulatory variables recorded (namely, wrist temperature and motor activity) permitted us to infer sleep/wake patterns (Ortiz-Tudela et al., 2010, 2014). The different delays found in sleep onset, midpoint and offset during the self-selected light/dark cycle stage highlights the importance of maintaining a regular light/dark cycle and daylight exposure, as well as other non-photic time cues (the participant was socially isolated and did not have knowledge of time) to entrain the circadian system. The delay in sleep timing during the constant dim light conditions reflects the natural tendency to delay and free-run in the absence of cyclic environmental and social cues, revealing an underlying clock/τ longer than 24 h (also found in the other variables studied). This has been previously described in the literature, both in constant dim light experiments (but with knowledge of clock time) and in forced desynchrony (FD) protocols (Middleton et al., 1996; Hiddinga et al., 1997; Carskadon et al., 1999; Czeisler et al., 1999; Burgess and Eastman, 2008). However, as far as we know, this is the first time that τ has been derived in a number of variables under constant dim light in conditions of both social and temporal isolation.

Apart from the circadian component of sleep, the homeostatic component of sleep was also observed in this experiment. The relatively long sleep duration exhibited by the participant at the beginning of the experiment likely reflects the typical sleep debt occurring under normal daily life conditions (Bonnet and Arand, 1995; Rajaratnam and Arendt, 2001; Basner et al., 2007) that recovers/lengthens when there are no commitments or obligations.

Regarding psychomotor performance, we found a clear diurnal pattern in reaction time at the beginning of the study, with better performance during the daytime. However, both reaction time and DSST (a test for memory/concentration) showed a tendency to improve throughout the study. This tendency is most likely due to a learning/practice effect (Beres and Baron, 1981), since a limitation of this study was the lack of intensive training on the different tests before the experiment. The dampening found in the day-night difference in the dim light conditions was unlikely due to the constant dim light conditions *per se*, since fast reaction times were not altered but were even faster, while slower reaction times under the light/dark conditions were clearly reduced, probably as a result of the already mentioned training effect. However, this reduction in amplitude would also be in agreement with that exhibited by wrist temperature, which would confirm the described relationship between core body temperature and performance as measured by psychomotor vigilance or code substitution tests (Johnson et al., 1992; Wright et al., 2002). In early work, Kleitman et al. (1938) postulated a causal role for body temperature on performance, although more recent studies suggest that cognitive performance is more complex and is influenced by a variety of factors other than only external and internal changes in body temperature [reviewed in Schmidt et al. (2007)]. Wrist temperature, for its part, has been proposed to be a good predictor of sleepiness (Sarabia et al., 2008; Madrid-Navarro et al., 2018) and the evening onset of melatonin synthesis (*dim light melatonin onset*, DLMO) (Bonmati-Carrion et al., 2013), thus peripheral wrist temperature could also be an objective way to predict the effect of time of day on cognitive performance, since sleepiness and cognitive performance have been reported to be closely related (Dinges et al., 1997). By contrast, results from the DSST did not show a clear daily pattern during any part of the study.

Physical performance also followed a diurnal rhythm with both grip strength and sprint speed exhibiting better results in the afternoon and following an inverse relationship with wrist temperature. Indeed, the circadian rhythm for physical performance has previously been found to be closely related to core body temperature [which follows an inverse pattern with that of peripheral temperature (Kräuchi and Wirz-Justice, 1994)], which has been suggested to increase energy metabolism, improve muscle compliance and facilitate actin-myosin cross-bridging [reviewed in Teo et al. (2011)]. Here we demonstrate that peripheral temperature does not seem to be directly contributing to better physical performance. Rather physical performance appears to be a reflection of sleepiness [also reflected by peripheral temperature (Kräuchi et al., 1997)] thus increased sleepiness, higher wrist temperature and worse performance.

Mood is another aspect of psychological wellbeing, which also followed a diurnal pattern, showing more depressive moods toward the night. Our results also indicate a clear relationship between mood and sleepiness, as previously suggested (Johnson et al., 1990; Dinges et al., 1997; Settineri et al., 2010, 2012; Wong et al., 2013). The observed dampening of the amplitude of this rhythm under the constant dim light conditions would suggest a possible masking effect of the light/dark cycle on the daily mood rhythm. Mood can also affect cognitive and physical performance, being suggested as an indirect way for sleep to affect both performance types (Wong et al., 2013).

The last part of this study comprised simulation of an early shift work schedule (light/dark cycle with early wake up), producing misalignment between the biological clock and desired sleep/wake (Möller-Levet et al., 2013; Eckel et al., 2015). The observed sleep parameters were those expected in this kind of schedule, with an advance in the mid-sleep because of the forced early waking, followed by an earlier sleep onset due to the increased homeostatic pressure. A limitation of this study, however, is the short duration of this stage of the experiment, not being long enough to assess the long term effect of this simulation on circadian rhythmicity. Another effect of the light/dark cycle with early wake up was the acute decline in the majority of performance variables, which could be due to the acute effect of sleep restriction, sleep inertia or that the assessment was performed at a different circadian phase. This performance impairment due to awakening during the biological night has also been previously shown (Scheer et al., 2008).

One limitation of this study regarding light exposure was use by the participant of a video-camera with a light-emitting screen. Although set at minimum brightness (the illuminance was not measured), these devices emit short wavelength-enriched light, the most active on the circadian clock [reviewed in Bonmati-Carrion et al. (2014)]. Although our results suggest that the synchronizing effect of this intermittent, low light during the dim light phase would be minimal, we cannot rule out this possibility.

With the evident limitation of a single case study, this experiment, originally designed as part of a television documentary, has also served as a case study of time and social isolation to show how different circadian rhythms persist and free-run with different periodicities under constant dim light conditions. The acute effect of an early start/sleep curtailment shift work simulation on performance has also been demonstrated. Confirmation of these results in future experiments is warranted.

## Data Availability Statement

The datasets generated for this study are available on request to the corresponding author.

## Ethics Statement

All research protocols were approved by the BBC’s compliance department, who reviewed the experiment design and ethical issues. All research was performed in accordance with relevant guidelines and regulations. A full risk assessment was carried out by the documentary producers, that was reviewed by an independent external risk assessment company (First Option – health and safety specialists who advise the television industry). The participant provided their written informed consent to participate in this study and the participant was aware that he could withdraw from the experiment at any time. The participant also underwent an independent psychological assessment to ensure he was a suitable subject for the experiment. The participant was also offered follow up support if needed.

## Author Contributions

All authors conceived and designed the study and reviewed the manuscript. VR, TC, and TW acquired the data. M-AB-C and JM analyzed the data. M-AB-C wrote the manuscript, with contributions from DS, VR, M-AR, and JM, and prepared the figures.

## Conflict of Interest

M-AR and JM are founding partners of Kronohealth SL, a spin-off company (co-founded by the University of Murcia) that has not contributed financially to this study. VR is a scientific advisor to Lumie Ltd. TC and TW were employed by Windfall Films, London. The remaining authors declare that the research was conducted in the absence of any commercial or financial relationships that could be construed as a potential conflict of interest.

## Footnotes

1 https://kronowizard.um.es/
