# Supplementary figures and images for: Living Without Temporal Cues: A Case Study
Source: Front Physiol. 2020 Feb 7;11:11. doi: 10.3389/fphys.2020.00011 (PMC7020909; doi:10.3389/fphys.2020.00011)

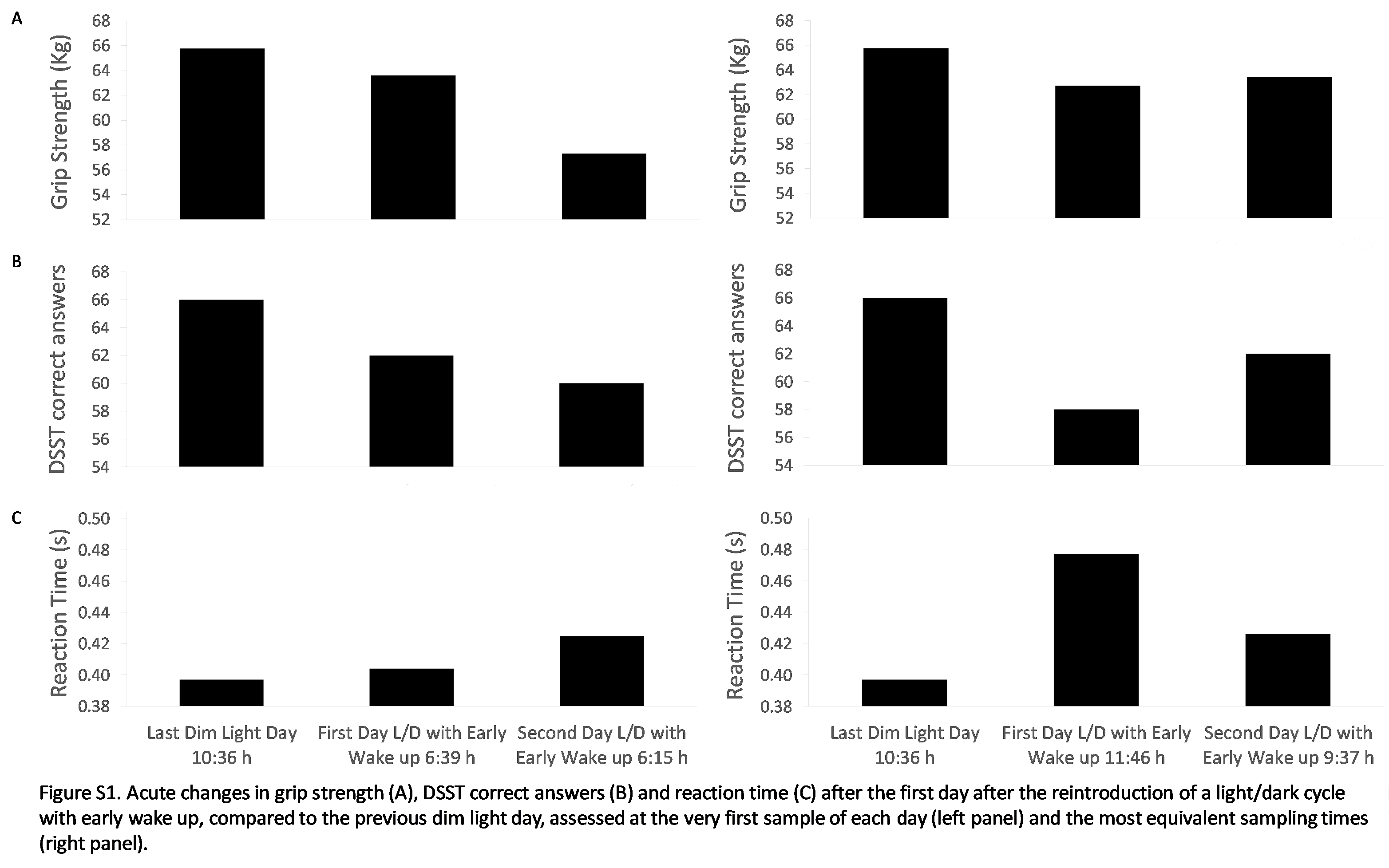

Supplement: Supplementary file 1 [file Image_1.tif]
